# Usefulness of Different Pathological Scores to Assess Healing of the Mucosa in Inflammatory Bowel Diseases: A Real Life Study

**DOI:** 10.1038/s41598-017-07338-x

**Published:** 2017-07-28

**Authors:** Vincenzo Villanacci, Elisabetta Antonelli, Francesco Lanzarotto, Anna Bozzola, Moris Cadei, Gabrio Bassotti

**Affiliations:** 1 Pathology Section, Department of Molecular and Translational Medicine, Brescia, Italy; 2 Gastroenterology Section, 1st Medical Clinic, Spedali Civili and University of Brescia, Brescia, Italy; 3 Gastroenterology Unit, Perugia General Hospital, Perugia, Italy; 4 Gastroenterology and Hepatology Section, Department of Medicine, University of Perugia School of Medicine, Perugia, Italy

## Abstract

The concept of remission for patients with inflammatory bowel diseases has recently evolved, and should also include histological healing of the mucosa, difficult to evaluate since there is no agreement on pathological scores and those available are quite complex to use in the daily routine. We evaluated the possible usefulness of a simplified pathological score to assess histological healing of the mucosa in inflammatory bowel diseases patients compared with four commonly proposed pathological scores. Slides from 24 patients (12 Crohn’s disease, 12 ulcerative colitis, age range 24–62 years), pre- and post-treatment with biological agents and displaying endoscopic remission were assessed by two pathologists. Pre- and post-treatment results and the time employed to calculate the various scores were obtained. All scores were useful to document highly significant post-treatment decreases of histological activity. However, the simplified score needed significant less time to be calculated for each slide, had high inter-rater agreement, and avoided subjectivity from the pathologists. The simplified score is easy to calculate and seems apt to document histological healing of the mucosa, in a manner similar to the more complex scores. It remains to be established whether this score could simplify the daily routinary practice in this context.

## Introduction

In recent years, the introduction in the clinical arena of inflammatory bowel diseases (IBD) of several and new therapeutic approaches, particularly based on biologic agents, has effectively changed the perspectives of both patients and physicians. In particular, there had been an evolution of the concept of treatment goals, shifting from the traditional one of clinical remission to a combination of clinical remission, laboratory normalization, and mucosal healing, the so-called “complete deep remission”^1^. Of interest, the concept of “deep remission” is evolving, and in the future will probably include the histological healing aspects in both Crohn’s disease (CD) and ulcerative colitis (UC) patients^2^, since it is increasingly clear that endoscopic remission is not necessarily paralleled by histological healing of the mucosa^3^.

However, while clinical remission and laboratory normalization are relatively easy to assess, the evaluation of mucosal healing is far more complex and difficult. In fact, notwithstanding the availability of several endoscopic techniques that are safe, feasible and effective for a detailed assessment of mucosal inflammation^4^, the use of endoscopic scores is presently hampered by their complexity for a routine use in clinical practice, by the lack of adequate interobserver agreement, and of a formal validation^5, 6^. Concerning histological healing of the mucosa things are even worst, in that there are presently available in literature a total of 22 different histological scoring systems for IBD (18 only for UC^7^), with only one or two fully validated^8^, and the microscopic features associated with IBD are considerably modified by the course of the disease and the treatments adopted^9^. Besides, things are further complicated by the fact that many of these scores are complex, extremely subjective in the interpretation of histological variables, and almost all of these are used for scientific purposes. Thus, to date we are unaware of the actual importance/usefulness of histological IBD scores in real-life situations.

Purpose of the present study was to assess the handiness of four published histological scores^10–13^, and to compare them with a simplified one, to evaluate their possible usefulness in the evaluation of histological healing of the mucosa in IBD patients in the daily routine.

## Results

**Figure 1:**
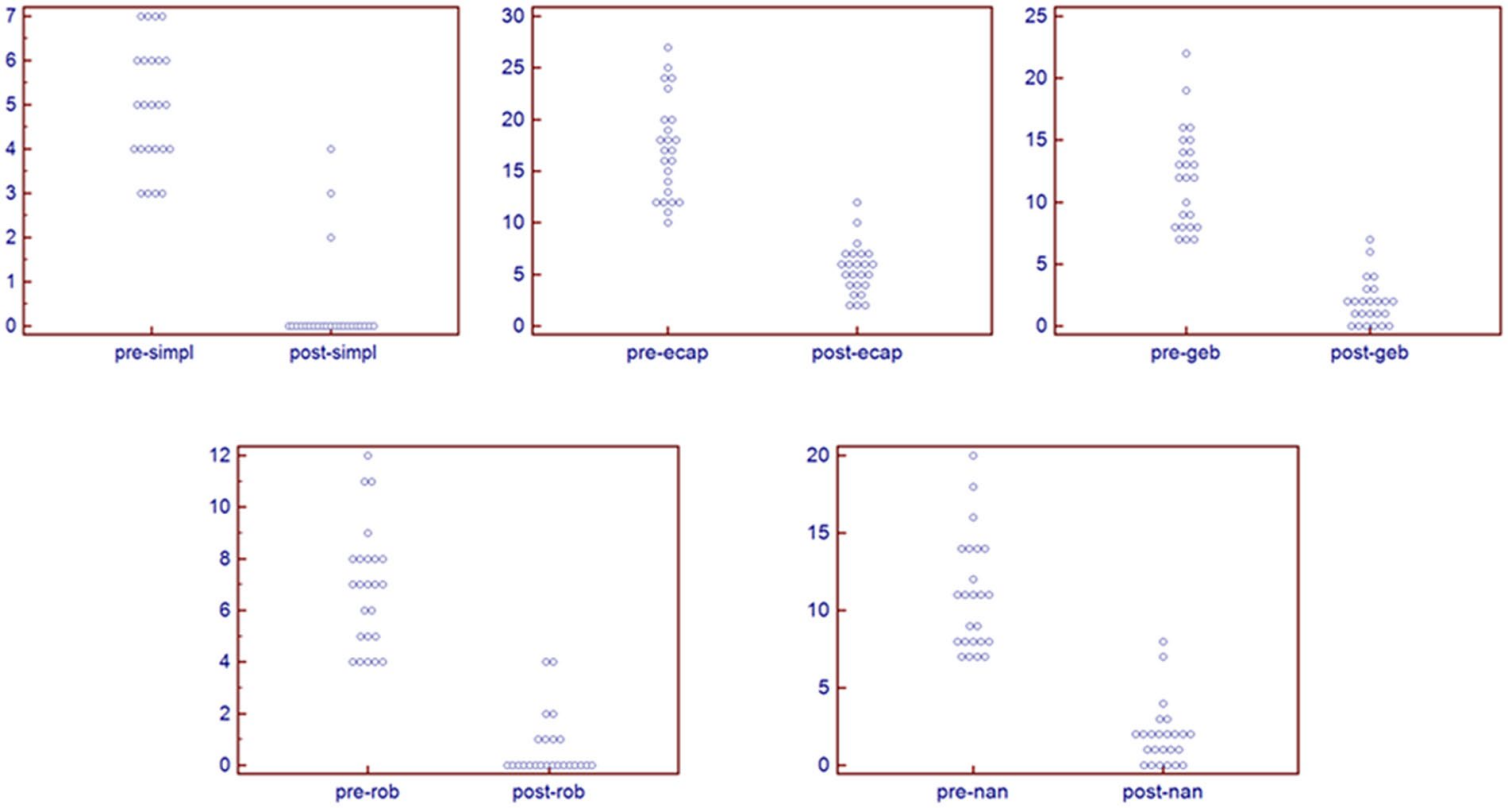
Individual values, pre- and post therapy, of the various scores utilized.

**Figure 2:**
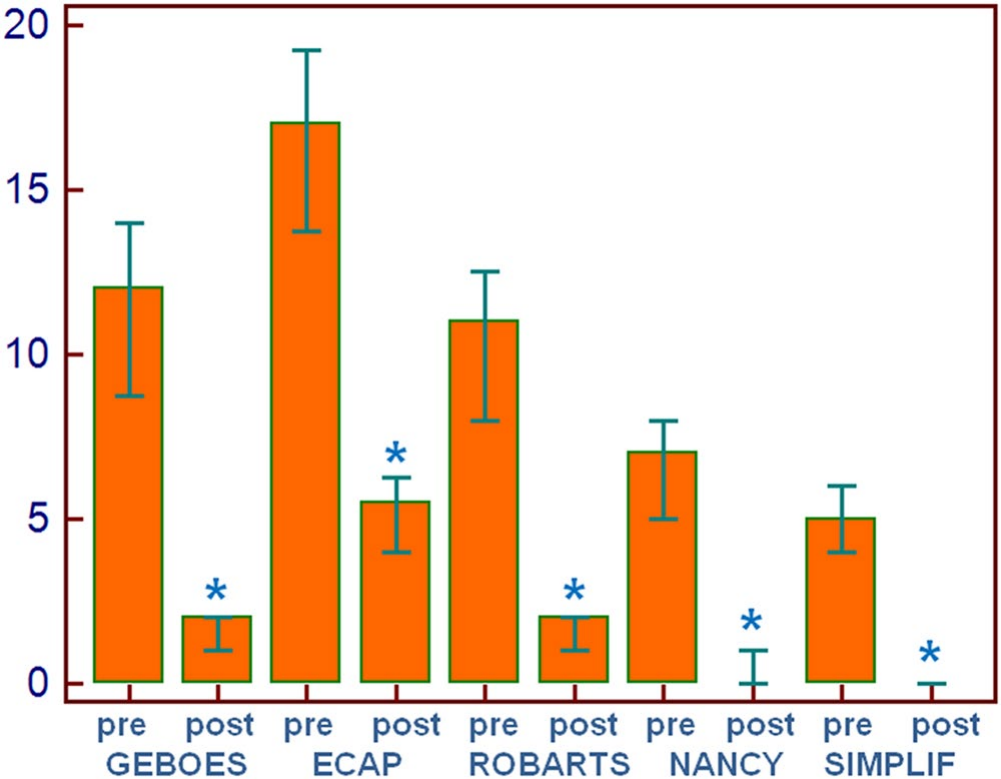
Pre- and post-therapy assessment with the various scores utilized.

Demographic, clinical and endoscopic variables are shown in Table 1. Pathological assessment with the various scores is shown in Table 2. All post-therapy scores demonstrated highly significant decreased values compared with the basal state (active disease) (Fig. 1). On average, a decrease of the basal score of more than 2/3 of the basal values was observed for each system (Fig. 2), suggesting that all scoring systems were similarly useful to assess histological healing of the mucosa. Inter-rater agreement was very good for the various scores (weighted Kappa = 0.94 for the simplified score, 0.93 for the ECAP score, 0.93 for the Geboes score, 0.91 for the Robarts score, and 0.95 for the Nancy score).

**Table 1:** Demographic, clinical, and endoscopic variables of the patients under investigation. Data are presented as medians (95% CI).

Age	37.5 (32.7–43.9)		
Sex	9 M, 15 F		
Diagnosis	Ulcerative colitis: 12		
	Crohn’s disease: 12		
PMS	pre-therapy	post-therapy	p
	5 (2–8)	0 (0–0)	0.0005
HBI	pre-therapy	post-therapy	p
	5 (4–6)	0.5 (0–1)	0.0005
S-IBDQ	pre-therapy	post-therapy	p
	159 (136–177)	203 (198–212)	0.0002
SES-CD score	pre-therapy	post-therapy	p
	11 (7–17)	1 (0–3.8)	0.0005
Mayo endoscopic score	pre-therapy	post-therapy	p
	2.5 (2–3)	0.5 (0–1)	0.0005

Abbreviations: HBI = Harvey-Bradshaw Index; PMS = partial Mayo score; SES-CD = simplified endoscopic score for Crohn’s disease; S-IBDQ = short inflammatory bowel disease questionnaire.

**Table 2:** Pre-and post therapy histological scores to assess histological healing of the mucosa in patients with endoscopic remission. Data are presented as medians (95% CI).

Score	Basal (experienced pathologist)	Basal (moderately experienced pathologist)	Post-therapy (experienced pathologist)	Post-therapy (moderately experienced pathologist)	p value compared to basal (for both pathologists)
Geboes (range 0–20)	12 (8.75–14)	12 (9–14)	2 (1–2)	2 (0.7–2)	<0.0001
ECAP (range 0–29)	17 (13.75–19.25)	17 (14–20)	5.5 (4–6.25)	5.5 (4.8–7)	<0.0001
Robarts (range 0–12)	7 (5–8)	7 (5–8)	0 (0–1)	0 (0–1)	<0.0001
Nancy (range 0–21)	11 (8–12.5)	11 (8–14)	2 (1–2)	2 (1–2)	<0.0001
Simplified (range 0–8)	5 (4–6)	5 (4–6)	0 (0–0)	0 (0–0)	<0.0001

**Figure 3:**
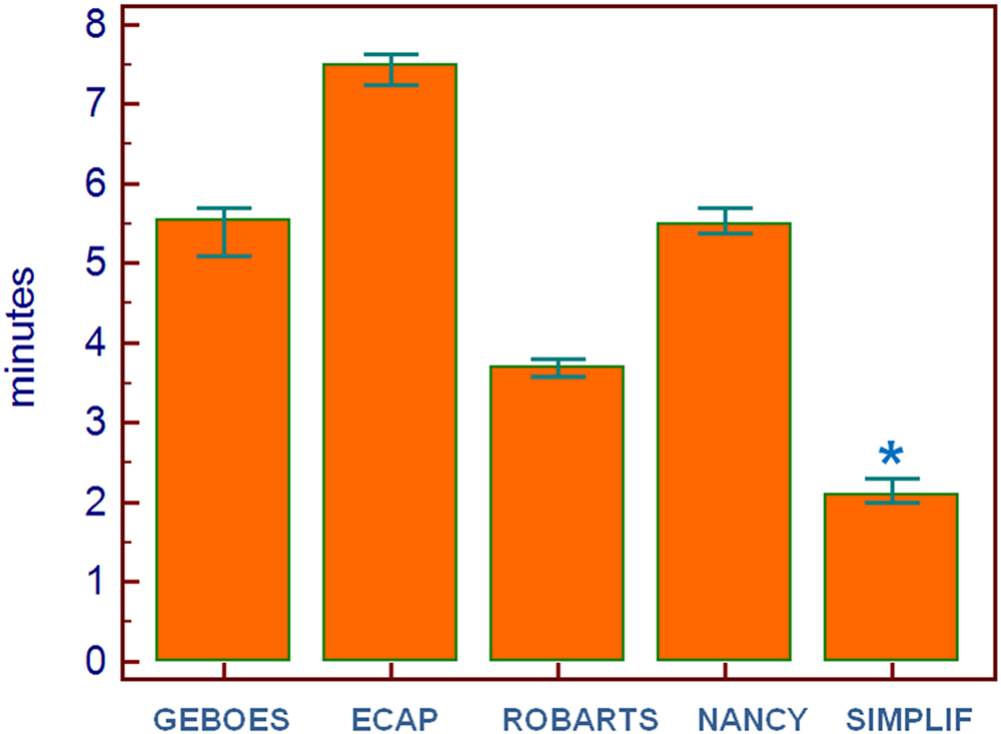
Time (in minutes) needed to calculate the various histologic scores.

Table 3 shows the time needed to assess the various scores. The moderately experienced pathologist took significantly more time to evaluate the slides (p < 0.001 for each score); however, for both pathologists the Friedman’s test showed that there were significant differences between scores (F = 508.9, p =  < 0.001), with the ECAP requiring significant more time and the simplified score needing a significant less amount of time (actually, almost half) to evaluate the slides, compared to the other scores (Fig. 3).

**Table 3:** Time (in minutes) needed to calculate the various pathological scores. Data are presented as medians (95% CI). Data are presented as medians (95% CI).

Score	Experienced pathologist (mins)	Moderately experienced pathologist (mins)	p
Geboes (range 0–20)	5.5 (5–5.7)^b,c^	6.5 (6–6.7)^b,c^	^b^0.0001 vs ECAP, Robarts and simplified; ^c^ n.s. vs Nancy
ECAP (range 0–29)	7.5 (7.2–7.6)^a^	8.5 (8–9)^a^	^a^<0.0001 vs all other scores
Robarts (range 0–12)	3.7 (3.6–3.8)^d,e^	4.6 (4.5–5)^d,e^	^d^0.0001 vs Nancy and simplified; ^e^n.s. vs Geboes and ECAP
Nancy (range 0–21)	5.5 (5.4–5.7)^e,f^	7 (6.6–7.3)^e,f^	^e^n.s. vs Geboes and ECAP; ^f^0.0001 vs Robarts and simplified;
Simplified (range 0–7)	2.1 (2–2.3)^a^	3 (3.2)^a^	^a^<0.0001 vs all other scores

## Discussion

To date, the concept of “remission” for patients with IBD has evolved from mere clinical aspects and includes endoscopic remission, in addition to clinical remission and laboratory normalization^1, 14, 15^. However, it is a matter of fact that an endoscopic remission is not always paralleled by a histologically quiescent disease^16–18^, and that obtaining both endoscopic and histological healing provides the best chances for a sustained remission^19^.

The limiting problems of this approach are limited by the fact that: (a) endoscopic scores are quite complex, lack a formal validation, and their reproducibility in IBD remains suboptimal, which could potentially have major effects on therapeutic choices^5, 6, 20^; (b) there are numerous histological scores (only a couple fully validated), strongly influenced by treatments, and often complex to use in clinical practice^7–9^.

Concerning assessment of histological healing of the mucosa in IBD, we feel that the availability of 22 different scoring systems present in literature represents a significant obstacle toward homogenization; besides, these scoring systems are almost always utilized in the context of clinical research trials, and things in real life may be quite different. In fact, studies from real life conditions suggest that the diagnostic prerequisites for a diagnosis of IBD are often unfulfilled^21^, and we have repeatedly proposed a simplified approach for histological evaluation of IBD patients in the daily routinary practice^3, 9, 22^. We feel that this simple approach, as in this study, can be utilized to assess histological healing of the mucosa in both CD and UC in real life conditions.

On the above basis, in the present study we compared a simplified score to assess histologic mucosal healing with four commonly used scores; although all scores were similarly useful to detect histological healing of the mucosa in patients with macroscopically (i.e., endoscopic) documented remission, the simplified score required a significant, considerable (almost the half) less time to be calculated for each slide, since the morphological variable to evaluate are extremely simple in routine practice for every pathologist. Thus, by considering an average of two correctly oriented biopsies, the amount of time required for a single patient was approximately five minutes with the simplified score, but it could treble with the more complex one. Besides, the simplified score introduced less subjectivity (and therefore, less bias) in the assessment of histologic features, based also on a precise location of the activity/inactivity of the disease along the terminal ileum and the different colonic segments. Moreover, in yielding significant results for the assessment of histological healing as the more complex scores, it could be hypothesized that the simplified score might be useful for the daily practice also in low-volume or less experienced pathology units. Under this light, we observed a very good inter-rater agreement although, as it could have been expected, the moderately experienced pathologist needed significant more time to read the slides.

Some points merit to be discussed. First, the presence of neutrophils in the crypts (with the subsequent development of crypt abscesses) and in the lamina propria should be considered as the actual markers of disease activity, similarly to what happens in other gastrointestinal inflammatory diseases. For instance, in the stomach the presence of neutrophils aggressive on the crypts is considered as the morphological sign of an active gastritis^23^. Second, as indicated by the recent ECCO ESP statements^24, 25^, during the histological evaluation of colonic mucosa with morphological features suggestive of IBD the presence of basal plasma cells has a high predictive value for the diagnosis of IBD and it is considered an important marker for the differentiation with other forms of colitis. We recently reported that this feature, in addition to the presence of eosinophils intermingled with basal plasma cells in the same anatomical position, has a high predictive value for the first diagnosis of IBD and it is present in all phases of the disease, either active or quiescent, as a marker of an IBD^26^. Thus, we feel that requiring the absence of basal plasma cells for “mucosal healing” is contradictory, because the presence of basal plasma cells in this phase is a sign of a preexistent IBD. Third, similarly to plasma cells, eosinophils are present in varying amounts in all phases of the disease, either in active or in quiescent colitis^27^, as we recently demonstrated^26, 28^. For this reason it is impossible to consider these cells as an indicator of disease activity. Fourth, the need of a correct methodological approach, in addition to the availability of exhaustive clinical and endoscopic data, is of paramount importance, as also stressed by the recent ECCO ESP statements (“For a reliable diagnosis of IBD, ileocolonoscopy rather than rectoscopy should be performed. A minimum of two biopsies from at least five sites along the colon, including the rectum and the terminal ileum, should be obtained and possibly correctly oriented on acetate cellulose filters”)^24^.

Of course, this study has several limitations. For instance, the study group was a selected one, treated only with a biologic agent to minimize as much as possible the interferences of various drug regimens. Moreover, although we found it very useful at least for the common daily practice, the simplified score has not been formally validated. In addition, the evaluation of the slides was carried out by gastrointestinal pathologists, quite familiar with the various scoring systems; in more common conditions in which the pathological assessment (as often happens) is made by general pathologists the use of complex scores may require much more time and/or introduce further biases in the interpretation of results.

More detailed scores would be more apt or more precise for scientific purposes, and result in a better definition of histological healing of the mucosa, even though to date is difficult to image a standardization of this variable, due to the numerous scores proposed and the lack of validation. On the other hand, this study tried for the first time to propose a score useful to effectively assess histological healing of the mucosa in IBD, including CD.

In conclusion, although the road toward an optimal assessment of therapeutic results in IBD patients is still long and winding, we feel that some improvements to simplify the evaluation of histological healing might be introduced, at least for the daily practice. Indeed, there is some recent acknowledgment that the proposed scores are too complex to be used in clinical practice, and attempts toward simplification are ongoing^29^. Whether the simplified score could be useful for this purpose, or even in clinical trials, remains to be established, although we feel that simplification may eventually led to a more fruitful approach to evaluate the therapeutic efficacy in IBD patients.

## Methods

Pathological slides (H&E) from 24 IBD patients (12 CD with ileocolonic involvement, 12 ulcerative pancolitis, age range 24–62 years), pre- and post-treatment with biological agents and in clinical remission, were retrieved from our archives after calculating that a minimum number of 6 subjects per group was needed with an α error of = 0.05 and a β error of 0.10. Biological agents utilized were infliximab (6 CD patients, all 12 UC patients) and adalimumab (6 CD patients); median duration of therapy was 15 (15–25) months for the entire group, 15 (15–34) months for CD and 18 (15–27) months for UC, respectively. Pre- and post-treatment clinical status and was assessed by means of the Partial Mayo Score (PMS) for UC^30^, the Harvey-Bradshaw index (BHI) for CD^31^, and the quality of life with the Short Inflammatory Bowel Disease Questionnaire (S-IBDQ)^32^.

**Figure 4:**
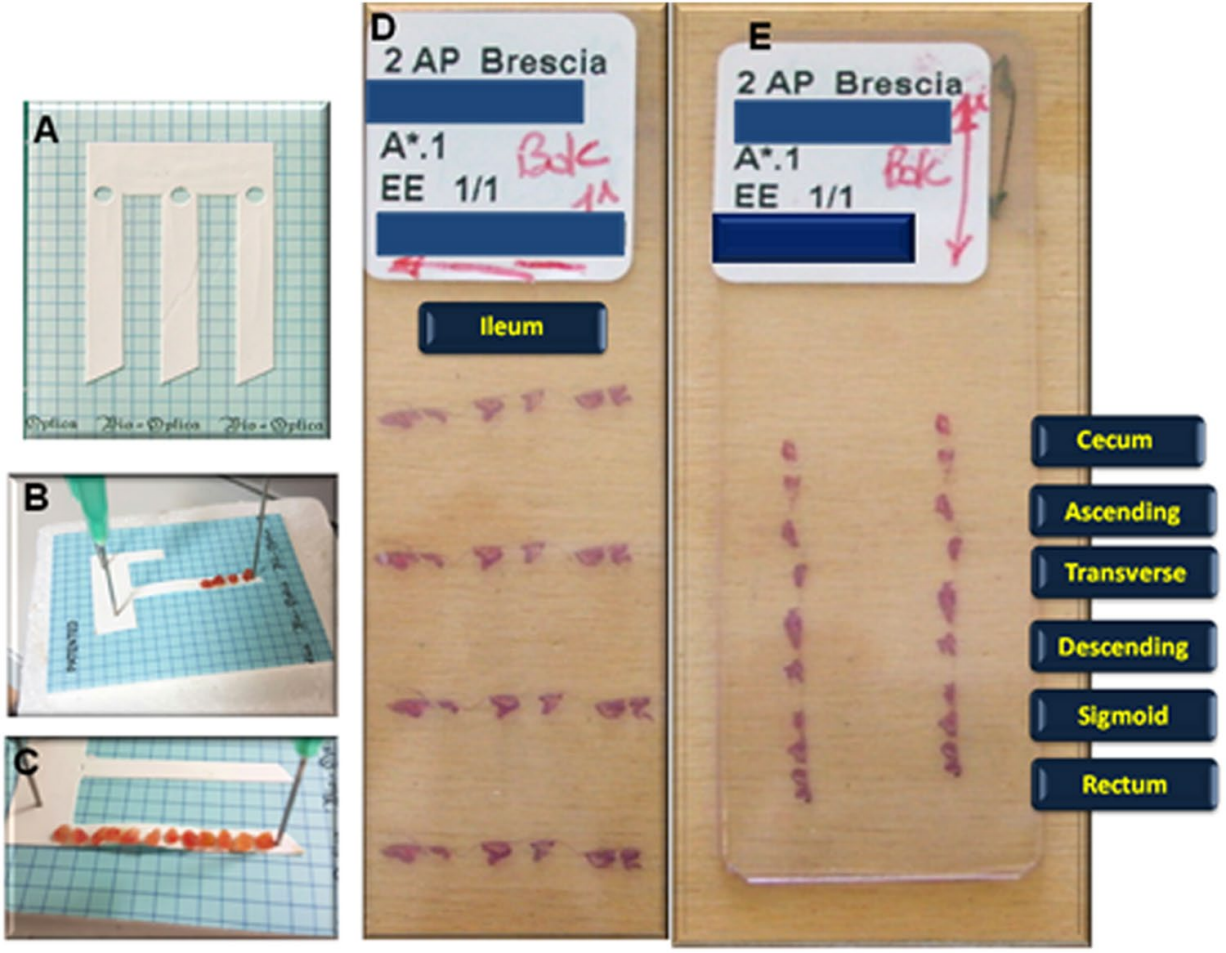
(**A–C**) Correctly orienting the biopsies on acetate cellulose filters; (**D** and **E**) correctly oriented ileal and colonic biopsies slides.

For the slides to be evaluated, the following conditions had to be met: (1) treatment with only a biologic agent; (2) biopsies correctly oriented on acetate cellulose filters (Fig. 4, A–C); (3) complete anatomical biopsy sampling according to the ECCO guidelines^24^ (at least four samples from the terminal ileum, and at least two samples from cecum, ascending colon, transverse colon, descending colon, sigmoid, and rectum) pre- and post-therapy (Fig. 4D,E). Thus, usually having three slides (one for terminal ileum, one from the cecum to the descending, one for the sigmoid and the rectum), a total of 144 slides (72 pre- and 72 post-therapy) was evaluated, with a total number of 384 biopsies examined; (4) post-treatment samples obtained from patients with endoscopic remission, according to the simplified endoscopic score for Crohn’s disease (SES-CD)^33^ and the Mayo endoscopic score (for UC)^34^.

**Figure 5:**
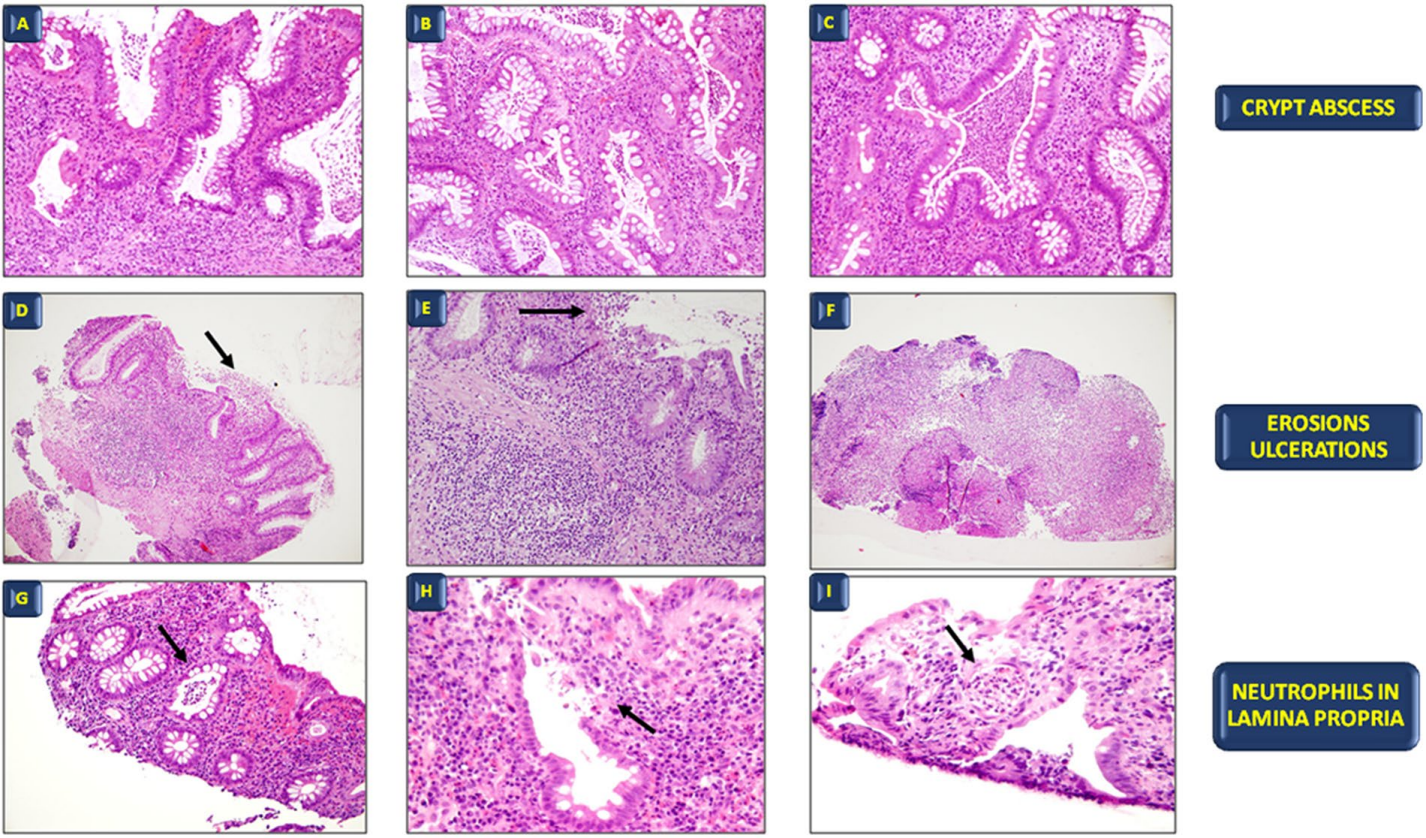
The three different morphological aspects of the simplified pathological score. (**A–C**) Crypt abscesses. (**H,E**), original magnification A, ×10; B, ×20; C, ×40. (**D–F**) Erosions and ulcers. (**H,E**), original magnification D, ×10; E, ×20; F, ×10. (**G–I**) Neutrophils in lamina propria; (**H,E**), original magnification G, ×10; H, ×20; I, ×40.

The slides were coded and blinded by one of the authors (MC) and read in blind by an experienced gastrointestinal pathologist (VV) and a moderately experienced pathologist (AB), who assessed them according to four previously published scores (Geboes^10^, ECAP^11^, Robarts^12^, and Nancy^13^). These scores were chosen among the numerous ones available in literature on the basis of being better known by pathologists in our country. In addition, a simplified score developed to assess mucosal healing in IBD (Table 4) and utilized in the daily routinary practice was calculated^35^. This score takes into consideration only three well defined histologic variables: (a) crypt abscesses; (b) presence of granulation tissue or aggregates of inflammatory elements in the superficial part of the mucosa, indicative of erosions or ulcers; (c) neutrophils in the lamina propria (Fig. 5) and their anatomic localization, thus greatly limiting the subjective interpretation by the pathologist. In particular, the presence of neutrophils was considered as the hallmark to differentiate between an active and a resolving/quiescent phase, as expression of therapeutic efficacy, i.e. histologic mucosal healing.

**Table 4:** Simplified pathological score utilized to assess histological healing in IBD patients.

1. crypt abscesses (presence of neutrophils aggressive on crypts):		◯presence	(1)
		◯absence	(0)
2. erosions/ulcerations (presence of granulation tissue):		◯presence	(1)
		◯absence	(0)
3. neutrophils in the lamina propria:		◯presence	(1)
		◯absence	(0)
4. site(s) of involvement			
	◯terminal ileum	◯active	(1)
		◯quiescent	(0)
	◯right colon	◯active	(1)
		◯quiescent	(0)
	◯transverse colon	◯active	(1)
		◯quiescent	(0)
	◯left colon	◯active	(1)
		◯quiescent	(0)
	◯rectum	◯active	(1)
		◯quiescent	(0)

Pre- and post therapy results were then obtained, as well as the time needed to evaluate the different scores for each slide.

Pre-and post-therapy results were compared by the Student’s t-test or the Wilcoxon test for paired samples after the D’Agostino-Pearson test ascertained whether the data were normally distributed or not. The time needed to evaluate the different scores was compared by the Friedman’s test. Values of p < 0.05 were chosen for rejection of the null hypothesis. Inter-rater agreement between the two pathologists were calculated by means of weighted (linear weights) Kappa statistic; values in the range 0.81–1 were considered as very good. Data are presented as medians (95% CI).

Since this was a retrospective study, no individual patient identification was involved and no study-driven clinical intervention was performed; therefore, our IRB waived formal review and approval, deeming the study to be an extension of existing procedures.
